# A case of *Talaromyces marneffei* infection that required differentiation from *Pneumocystis* pneumonia

**DOI:** 10.1016/j.idcr.2022.e01654

**Published:** 2022-12-01

**Authors:** Mieko Tokano, Norihito Tarumoto, Kazuo Imai, Jun Sakai, Masahiro Kodana, Erika Naito, Yoshitaka Uchida, Makoto Nagata, Shigefumi Maesaki

**Affiliations:** a Department of Infectious Disease and Infection Control, Saitama Medical University, 38 Morohongo, Moroyamamachi, Irumagun, Saitama 350-0495, Japan; b Departments of Allergy and Immunology, Faculty of Medicine, Saitama Medical University, 38 Morohongo, Moroyamamachi, Irumagun, Saitama 350-0495, Japan; c Department of Clinical Laboratory, Saitama Medical University, 38 Morohongo, Moroyamamachi, Irumagun, Saitama 350-0495, Japan; d Department of Respiratory Medicine, Saitama Medical University, 38 Morohongo, Moroyamamachi, Irumagun, Saitama 350-0495, Japan

**Keywords:** *Talaromyces marneffei*, *Pneumocystis pneumonia*, Human Immunodeficiency Virus (HIV), Acquired immunodeficiency syndrome (AIDS), Voriconazole, Japan

## Abstract

We report a case of *Talaromyces marneffei* fungemia in a patient with HIV infection with a history of travelling to southern China. At first, *Pneumocystis* pneumonia was considered in this case because chest CT images showed typical ground-glass opacity and elevated β-D-glucan levels. However, PCR testing of sputum for *Pneumocystis jirovecii* was negative and a filamentous fungus was isolated from blood cultures. The cultured fungus was subsequently identified as *T. marneffei*, and the patient was considered to have pneumonia caused by this organism. However, skin disease and lymphadenopathy, which are common in T. marneffei infections, were not observed during the disease course. This patient was successfully treated with voriconazole and consequently the chest CT shadow disappeared. In the present case, *T. marneffei* infection required differentiation from pneumonia with *Pneumocystis jirovecii* infection.

## Introduction

*Talaromyces marneffei* (formerly *Penicillium marneffei*) is a thermally dimorphic fungus that causes disseminated infection among HIV-infected individuals. *T. marneffei* has a limited geographic distribution, mainly in Southeast Asia and southern China [1], [2]. Although this fungus is not native to Japan, more than a dozen cases of imported infection have been reported in the Japanese population. We here report a case of *T. marneffei* fungemia with HIV infection that required differentiation from *Pneumocystis* pneumonia.

## Case

A 47-year-old Japanese man presented to a hospital with a two-weeks history of dry cough, a one-week history of dyspnea, and weight loss (17 kg over several months). He had no medical history of note except for mild hepatic dysfunction during an initial examination. He had no relevant family history, and no history of blood transfusion, smoking, or obvious animal contact. He was an occasional drinker and was a university lecturer. On admission, his consciousness was clear, and a physical examination revealed the following: body temperature, 37.2 °C; blood pressure, 104/66 mmHg; pulse, 111 beats/minute; and SpO_2_, 96% (face mask 7 L/min). Chest auscultation revealed no heart murmur or any crackles in the bilateral lung fields. Superficial lymphadenopathy, skin rash, and hepatosplenomegaly were absent.

**Fig. 1:**
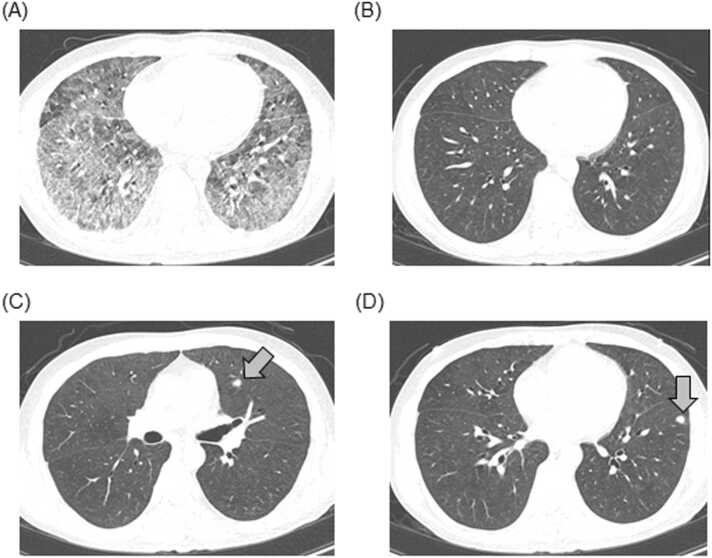
(A) Thoracic CT on admission showing diffuse ground glass opacification throughout both lungs. (B, C, D) Thoracic CT on the 21st day showed that the diffuse ground glass opacification had disappeared, but multiple nodules remained.

**Fig. 2:**
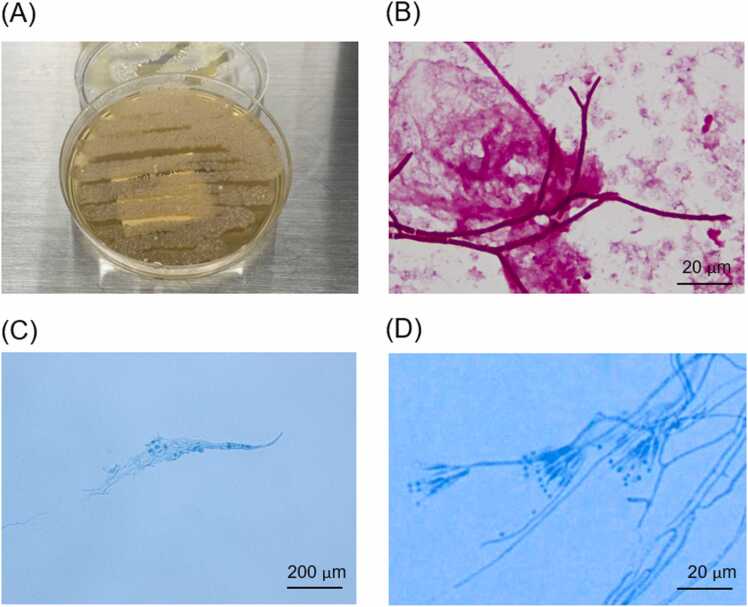
(A) Subculturing of the organism isolated from blood culture on Sabouraud agar medium after one week of incubation at 35 °C. (B) Gram staining from the patient's blood culture revealed the presence of filamentous fungi. (C, D) Lactophenol cotton blue staining showed fungi with broom-like bodies.

The examination findings on admission demonstrated (Table 1) a highly elevated β-D-glucan (BDG) level (550 pg/mL). The aspergillus galactomannan antigen detection in serum was negative. His HIV RNA titer was 8.5 × 10^6^ copy/mL and his CD (cluster of differentiation) 4^+^ T-cell count was 20 /μL. Therefore, he was diagnosed with HIV infection. Chest CT revealed diffuse ground glass opacification throughout both lungs except for the bilateral pulmonary apex and lung bases (Fig. 1A). Sputum culture only detected commensal bacteria. Blood culture was positive for two sets of aerobic bottles and Gram staining showed filamentous fungi. After one week of incubation at 35 °C on Sabouraud agar medium, white colonies were observed, and lactophenol cotton blue staining showed fungi with broom-like bodies. (Fig. 2A-D).

**Table 1:** Laboratory Findings at the First Presentation.

White blood cells	10200/μL	brain natriuretic peptide	35.6 pg/mL
Lymphocytes	1020/μL	Sialylated carbohydrate antigen KL-6	3311 U/mL
Neutrophils	8854/μL	β-D-glucan	550 pg/mL
Platelet	31.8 × 10^4^/μL	interferon-gamma release assays for tuberculosis	negative
Hemoglobin	10.4 g/dL	HBs antigen	negative
CD4^+^ T-cells count	20/μL	HCV antibody	negative
Aspartate aminotransferase	52 U/L	CMV antigen	negative
Alanine aminotransferase	38 U/L	HIV-1 Western blots	positive
Lactate dehydrogenase	799 U/L	HIV-2 Western blots	negative
Creatinine	0.97 mg/dL	HIV-RNA	8.5 × 10^6^ copy /mL
Blood urea nitrogen	21.4 mg/dL	PCR for *Pneumocystis jirovecii*	negative
Sodium	136 mEq/L	Urinary antigen test for *Streptococcus pneumoniae*	negative
Potassium	3.9 mEq/L	Urinary antigen test for *Legionella*	negative
C-reactive protein	22.75 mg/dL		

Abbriviations: CD, cluster of differentiation; HB, hepatitis B; HCV, hepatitis C virus; HIV, human immunodeficiency virus; RNA, ribonucleic acid; PCR, polymerase chain reaction.

**Fig. 3:**
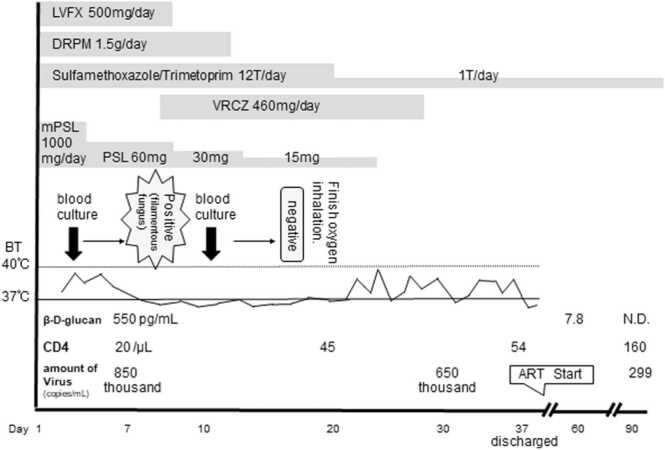
The course of treatment. The patient’s symptoms disappeared. LVFX, levofloxacin; DRPM, doripenem; VRCZ, voriconazole; mPSL, methylprednisolone; PSL, prednisolone; BT, Body temperature; N.D., not detected; ART, antiretroviral therapy.

PCR from sputum for *Pneumocystis jirovecii* was negative. However, the possibility of *Pneumocystis* pneumonia could not be ruled out clinically; thus, he was treated with sulfa/trimethoprim (ST) at a fixed dose of 9 mg/day and methylprednisolone pulse therapy (1000 mg/day for 3 days). In addition, doripenem (1.5 g/day) and levofloxacin (500 mg/day) were administered for possible complications of bacterial and atypical bacterial infections. The filamentous fungi detected in blood culture could not be determined using MALDI-TOF MS (Bruker, Germany, Filamentous Fungi Library 1.0). However, we administered voriconazole (VRCZ) for 3 weeks, considering the possibility of *Fusarium* spp. or *Aspergillus* spp. (Fig. 3). His respiratory status improved with the above treatment, and his BDG level decreased. Chest CT on the 21st day (Fig. 1B-D) showed that the diffuse ground glass opacification had disappeared, while multiple nodules remained. On day 22, his hypoxemia improved. The patient presented fever on day 24; this was thought to be the effect of the termination of corticosteroid treatment or drug fever. After 37 days of hospitalization, his symptoms disappeared, his general condition returned to normal, and his laboratory findings improved. On day 49, antiretroviral therapy (ART) was initiated. He started taking a fixed-dose tablet of dolutegravir, abacavir and lamivudine. At a 3-month follow-up examination, he appeared healthy with no symptoms and chest CT showed no abnormal findings. The treatment was successful and his symptoms did not recur.

We performed the next-generation sequencing for the unidentified microbe and its assembling was done. Briefly, *De novo* assembling using spades (https://cab.spbu.ru/software/spades/) was performed after discharge from the hospital based on the results of next-generation sequencing (iSeq 100 system, Illumina, USA). The internal transcribed spacer (ITS) region was matched to the National Center for Biotechnology Information (NCBI) nt/nr database using blastn. The ITS region of the cultured pathogen was perfectly (100%) identical to *T. marneffei* (NCBI accession no. CP045655.1). Furthermore, the whole genome sequence of *de novo* assembling was compared to the reference sequence of *T. marneffei* (NCBI accession no. CP045655.1) using the Average Nucleotide Index and confirmed to match 99.1% of the sequences. Based on these results, the fungus isolated and cultured from his blood culture was classified and identified as *T. marneffei*. In a subsequent interview he revealed that he had been employed in the People's Republic of Guangzhou, China for several years and that he had experienced sexual contact with a local woman several months before the onset of the disease.

## Discussion

*T. marneffei* infection is a deep-seated mycosis that is increasing in Southeast Asia and the southern region of People's Republic of China, and mainly occurs among HIV-infected individuals when the CD4^+^ T-cell count is < 50 /μL [1], [2]. After respiratory tract infection, the disease can disseminate hematogenously to multiple organs throughout the body, including the bone marrow and skin [1]. Representative symptoms include fever, weight loss, skin lesions, generalized lymphadenopathy, hepatosplenomegaly, and skin lesions with induration and ulceration are seen in approximately 80% of cases [3], [4], [5].

The diagnosis can be made by culturing various clinical specimens; however, the serodiagnostic and genetic diagnostic testing have not been put to practical use. Including our case, 13 cases of *T. marneffei* infection have been reported in Japan; 9 of these cases involved individuals with HIV infection and 7 of the patients had an elevated BDG level. Five had abnormal findings in the lung fields: nodular or infiltrative shadowing was seen on chest radiographs and/or CT scans. Only two cases showed ground-glass opacity. All cases had a history of travelling to endemic countries (Table 2). The present case certainly had a history of residence in the People's Republic of China. The first-line treatment is antifungal agents (e.g., amphotericin B lipid preparations or voriconazole), to which the pathogen is susceptible. Fortunately, the present patient was successfully treated with voriconazole.

**Table 2:** Reported cases of Talaromyces marneffei infection in Japan.

Age	Sex	National origin	Underlying disease	CD4^+^ T-cells counts /mm3	BDG pg/mL	Symptoms	Diagnostic method	Radiology findings	Medications	Outcome	Reference
38	m	Thailand	HIV	20	N.D.	fever, exanthema and lymphadenopathy	skin biopsy	abscess	FLCZ	death	[6]
22	m	Myanmer	HIV	1	elevated	exanthema, weight loss	skin biopsy	N.D.	N.D.	N.D.	[7]
41	f	Thailand	HIV	31	136	fever, lymphadenopathy, splenohepatomegaly	blood culture	diffuse miliary nodules	L-AMB→MCFG→ITCZ	recovered	[8]
30	m	Thailand	HIV	10	25.7	fever, general fatigue, cervical and subclavian lymphadenopathy	blood culture, lymph node culture	N.D.	L-AMB→ITCZ	recovered	[9]
35	m	India	HIV	60	N.D.	weight loss and skin lesions, exanthema	skin biopsy, lymph node biopsy	mediastinal and para-aortic lymphadenopathy	L-AMB→ITCZ	recovered	[10]
56	m	Thailand	Rheumatoid arthritis	N.D.	42.4	general fatigue, fever, loss of appetite, dyspnea, and sweating	BALF	diffuse miliary nodules with a random distribution	L-AMB	recovered	[11]
71	m	Thailand	Interstitial pneumonia	N.D.	normal range	coughing and dyspnea	BALF	bilateral ground-glass abnormalities	ITCZ	recovered	[12]
66	m	Vietnam	HIV	72	337	diarrhea, oral erosion, heartburn, and weight loss	bone marrow culture	mild splenomegaly and multiple enlarged lymph nodes at the hepatic hilum and peritoneum	FLCZ→ITCZ	recovered	[13]
50′s	m	Thailand	HIV	77	47.5	loss of appetite, weight loss and general malaise	blood culture, an aspirate from a supraclavicular lymph node	mild ground-glass opacities in a patchy distribution in both lungs	L-AMB	death	[3]
65	f	Vietnam	healthy individuals	N.D.	N.D.	poor appetite, weight loss, persistent non-productive cough. exanthema and intermittent fever	skin biopsy	N.D.	ITCZ	recovered	[14]
22	m	Vietnam	HIV	1	519.6	melena, hematemesis, lymphadenopathy, splenohepatomegaly and exanthema	skin biopsy, blood culture	N.D.	MCFG→L-AMB→ITCZ	recovered	[15]
27	f	Myanmar	HIV	18	under 6.0	headache consciousness disturbance	BALF, transbronchial lung biopsy	nodule	L-AMB→ITCZ	recovered	[16]
47	m	China	HIV	20	550	fever, coughing and dyspnea	blood culture	bilateral ground-glass abnormalities	VRCZ	recovered	This case

Abbreviations: CD, cluster of differentiation; BDG, β-D-glucan; N.D., no data; HIV, Human Immunodeficiency Virus; FLCZ, fluconazole; L-AMB, liposomal amphotericin B; MCFG; micafungin; ITCZ, itraconazole.

At first, *Pneumocystis* pneumonia was considered in this case because chest radiographs and CT images showed typical ground-glass opacity and the patient’s BDG level was elevated. Nevertheless, PCR testing of sputum for *P. jirovecii* was negative and a filamentous fungus was isolated from two sets of blood cultures. The cultured fungus was subsequently identified as *T. marneffei*, and the patient was considered to have pneumonia caused by this organism. However, skin disease and lymphadenopathy, which are common in *T. marneffei* infections, were not observed during the disease course. In addition, a nodular appearance was observed on chest computed tomography after treatment with voriconazole, suggesting that the early ground-glass opacity may have been associated with a marked decrease in the number of CD4^+^ T-cells in association with HIV infection.

However, since *T. marneffei* was not isolated from sputum in this case and we did not perform a microbial examination of bronchoalveolar lavage fluid or a histopathological examination of the lung lesions, it was not possible to further confirm whether the lung lesions were caused by *T. marneffei* infection. *T. marneffei* is a dimorphic fungus that becomes yeast-like at 37 °C and filamentous at 25–30 °C [5], which requires a laboratory with Biosafety Level 3 equipment, and further mycological testing—including an antifungal susceptibility test—was impossible in our laboratory.

In general, *T. marneffei* infection is not included in the AIDS indicator diseases in Japan; however, it is the third most common HIV-related opportunistic infection in Southeast Asia after cryptococcosis and tuberculosis [17]. *T. marneffei* has a limited geographic distribution, mainly in Southeast Asia and southern China, and disseminated *T. marneffei* infection can be fatal [18]. Therefore, inquiring about patients’ travel history is very important in HIV infection care. Since *T. marneffei* is not listed in the latest Bruker database, the species cannot be identified without using an in-house database, which can delay the diagnosis. Furthermore, because of the risk of laboratory-acquired infections, it is important to share clinical information with laboratory personnel. Our case suggests the importance of being aware of this specific infectious disease as a differential diagnosis in HIV-infected patients with a history of travelling to these regions.

## Funding

None.

## Ethical approval

Not applicable.

## Consent

Written informed consent was obtained from the patient for publication of this case report and accompanying images. A copy of the written consent is available for review by the Editor-in-Chief of this journal on request.

## Author contribution

M.T., N.T., K.I., J.S., E.N., Y.U., M.N., and S.M treated the patient. M.T., M.K, and N.T. erformed the next-generation sequencing for the unidentified microbe and its assembling was done. M.T., N.T., and S.M wrote the manuscript. All authors discussed the results and commented on the manuscript.
